# Editorial: Outcomes of stroke: prediction and improvement

**DOI:** 10.3389/fneur.2023.1256253

**Published:** 2023-07-25

**Authors:** Heling Chu, Longxuan Li, Bin Qiu, Yuping Tang

**Affiliations:** ^1^Department of Gerontology, Shanghai Sixth People's Hospital Affiliated to Shanghai Jiao Tong University School of Medicine, Shanghai, China; ^2^Department of Neurology, Ruijin Hospital, School of Medicine, Shanghai Jiao Tong University, Shanghai, China; ^3^School of Medicine, Yale University, New Haven, CT, United States; ^4^Department of Neurology, Huashan Hospital, Shanghai Medical College, Fudan University, Shanghai, China

**Keywords:** ischemic stroke, hemorrhagic stroke, prediction, outcomes, management, stroke, therapeutics

Stroke remains the second-leading cause of death and the third-leading cause of disability worldwide (1). Of the two types of strokes, ischemic and hemorrhagic, the former accounts for more than 80% of strokes, while the latter is more disabling and fatal. Despite ongoing efforts to explore effective management and prevention strategies, the annual number and burden of stroke continue to grow worldwide, especially in low- and middle-income countries (2).

More and more attention has been paid to strategies that result in favorable outcomes for all types of strokes. Multiple therapies, including thrombolysis, mechanical thrombectomy, and surgical intervention, are used in clinical practice (3, 4). Despite the improvement in predictive ability, the choice of these therapies may be influenced by multiple factors, namely the time windows and may possibly bring out unpredictable adverse effects. Therefore, it will be more helpful if a predictor can rapidly identify the risks that affect the outcome and treatment effectiveness after stroke (5, 6). The management strategies chosen according to the effective prediction may provide more clinical benefits.

This Research Topic, entitled “Outcomes of Stroke: Prediction and Improvement,” aims to investigate the predictors and effective management strategies that can predict and improve stroke outcomes. It consists of 37 articles, and a brief description of the research findings follows.

As ischemic stroke accounts for more than 80% of strokes, most of the articles deal with the predictors and therapeutic strategies of ischemic stroke. Some of the works explore useful predictors of the outcomes of ischemic strokes. Liu et al. demonstrated that the ratio of vascular endothelial growth factor to CBP/P300-interacting transactivator with Glu/Asp-rich C-terminal domain 2 (VEGF/CITED2) from peripheral blood mononuclear cells is an independent protective factor and has a potential predictive value in the collateral circulation of acute ischemic stroke (AIS) evaluated by diffusion-weighted imaging (DWI)-Alberta Stroke Program Early CT Score (ASPECTS). Liu et al. showed that the ratio of serum uric acid to serum creatinine (SUA/SCr) is negatively associated with the risk of early neurological deterioration (END) within 1 week in patients with branch atheromatous disease stroke. Another study also investigated biomarkers of END. Wang Z. et al. detected plasma neurofilament light chain (pNFL) via a novel ultrasensitive single-molecule array and found that pNFL is a promising biomarker of END in minor stroke with large vessel occlusion. Meanwhile, Ag Lamat et al. illustrated how electroencephalogram (EEG) abnormalities are associated with stroke type and imaging characteristics. Additionally, NIHSS score and anterior circulation stroke can be considered predictors of focal EEG slowing.

Recurrent ischemic stroke (RIS) is associated with increased mortality and a poor outcome. Chung et al. investigated gender differences and risk factors for RIS. In their study, they found that hypertension and dyslipidemia were significant risk factors in both genders. Risk factors that differed between genders are smoking and alcohol consumption in men and diabetes in women. Another study by Wang H. et al. from Fudan University developed a radiomics-based DWI prediction model that performed well in terms of predicting 1-year RIS. Another prediction model for RIS was developed by Elhefnawy et al. from real-world data. In this model, incorporating time into predicting the risk of RIS can positively contribute to predicting the prognosis of RIS. The risk of RIS changes with time after the index ischemic stroke. In addition to concomitant diseases, secondary prevention time also plays a vital role in predicting the risk of RIS in the population. The COVID-19 pandemic has had a great impact on the treatment of AIS. Gu et al. demonstrated that the pandemic exacerbates certain time delays and plays a significant role in early adverse outcomes in patients with AIS. Atrial fibrillation (AF) is a common cardiac arrhythmia that is associated with an increased risk of ischemic stroke. Lu M. et al. performed a meta-analysis showing that the neutrophil-to-lymphocyte ratio (NLR), as an important inflammatory indicator, is associated with a higher risk of stroke in AF patients. The incidence of stroke in AF patients with NLR ≥3 was 1.4 times higher than in those with NLR <3. A comparative analysis performed by Bem Junior et al. showed that decompressive craniectomy is currently the most effective measure to control refractory ICH in cases of malignant ischemic stroke. In addition, Kosugi et al. conducted a study to create a cortical infarction using photothrombosis over the motor cortex of non-human primates to establish a reproducible deficit in the reaching and grasping tasks. This research suggests that different recovery speeds for each movement may be influenced by the extent to which cortical control is required to properly execute each movement.

Intravenous thrombolysis using recombinant tissue plasminogen activator (r-tPA) is effective for the treatment of AIS, although uncertainties remain about the balance of benefits and risks in some circumstances. In their study, Yuan et al. demonstrated that the non-standard dose of r-tPA (0.6 mg/kg ≤ dose < 0.9 mg/kg) does not differ in safety and effectiveness compared with the standard dose (0.9 mg/kg). The findings suggest that, according to current evidence and guidelines, the standard dose should be regarded as the first choice, while the above non-standard dose could be an alternative option in the actual diagnosis and treatment process, considering the patient's clinical profile and financial condition. Meanwhile, Xu et al. evaluated the safety and efficacy of low-dose alteplase (0.55–0.65 mg/kg) compared with standard-dose (0.85–0.95 mg/kg) in Chinese patients with AIS using a real-world registry. The results indicate that low-dose alteplase has a significantly higher rate of death or disability at discharge without reducing the risk of symptomatic intracranial hemorrhage. The prediction of outcome after intravenous thrombolytic therapy was also included in our Research Topic. Jiang et al. showed that the difference in red blood cell distribution width from before to after rt-PA thrombolysis is an independent predictor of neurological outcome at 2 years after thrombolysis in patients with AIS. Fekete et al.'s work implies that older age, higher NIHSS, large vessel occlusion, and intra-arterial thrombolysis may correlate with intracranial hemorrhage after thrombolytic therapy, and such patients always have unfavorable outcomes in a prospective single-center study. Norata et al. demonstrated that the liver fibrosis-4 score is associated with poor 3-month outcome and symptomatic intracranial hemorrhage in AIS patients undergoing intravenous thrombolysis.

Endovascular thrombectomy has been shown to be effective in the treatment of acute ischemic stroke in patients with large vessel occlusion (7). Prediction of prognosis, especially in patients with successful revascularization, is critical. Shao et al. demonstrated that acidosis, including decreased $HCO_{3}^{-}$ and pH levels detected by arterial blood gas (ABG) testing, is associated with clinical outcomes after endovascular therapy. Post-thrombectomy intracranial hemorrhage (PTIH) is a serious complication of AIS following mechanical thrombectomy. Rossi et al. analyzed 122 thrombi from 80 AIS patients and detected S100b expression by immunohistochemistry. The results show that S100b is co-localized with neutrophils, macrophages, and T-lymphocytes in clots and that higher S100b expression is associated with PTIH. In the study by Wagner et al., 714 AIS patients received intravenous thrombolysis followed by endovascular thrombectomy. Their results suggest that shorter intervals between intravenous thrombolysis and endovascular thrombectomy are associated with better 3-month functional outcomes. To reduce in-hospital workflow time, Zhou T. et al. developed a one-stop stroke management (OSSM) platform, and in their study, the OSSM transfer model significantly reduced the in-hospital delay in AIS patients compared to the traditional transfer model. Acute basilar artery occlusion (BAO) is one of the most fatal diseases, with a high risk of mortality and disability. Luo et al. aimed to determine the most effective rescue measure for acute BAO after the failure of mechanical thrombectomy. They found that balloon angioplasty, Wingspan stenting, and Apollo stenting rather than Solitaire stenting could be considered successful and safe rescue options. Lu Y. et al. investigated the optimal type of anesthesia management for acute vertebrobasilar artery occlusion (VBAO). Their findings reveal that similar effectiveness and safety were observed between general anesthesia (GA) and non-GA during endovascular treatment for VBAO, and GA may provide better successful reperfusion for a worse presenting GCS score (≤8). Another study by Lee et al. also examined the effects of anesthesia management for endovascular thrombectomy on outcomes through a meta-analysis of randomized clinical trials and trial sequence analysis. The conclusion is that patients with acute anterior circulation ischemic stroke who receive GA are associated with a higher rate of successful recanalization and a better 3-month neurological outcome compared to those who receive conscious sedation. A systematic review and meta-analysis conducted by Duan et al. aims to evaluate the current evidence on the feasibility, efficacy, and safety of endovascular thrombectomy in patients with active cancer. They found that such patients are likely to have an unfavorable outcome and that active cancer may increase the risk of mortality after endovascular thrombectomy. Another meta-analysis by Wu et al. revealed that the above-mentioned inflammatory marker NLR tested at admission and post-treatment can be used as a cost-effective and readily available biomarker to predict 3-month poor functional outcome, symptomatic intracerebral hemorrhage (sICH), and 3-month mortality in AIS patients undergoing reperfusion therapy. Hemorrhagic transformation is one of the most devastating complications of reperfusion therapy in AIS patients. A meta-analysis by Sun et al. identified several predictors of hemorrhagic transformation, including atrial fibrillation, a higher NIHSS score, older age, higher serum glucose levels, the number of thrombectomy procedures, and lower ASPECTS.

ICH accounts for ~10%−20% of all stroke subtypes with high mortality, and the survivors always have a high degree of residual disability. Hematoma expansion (HE) is often observed in the early stages of ICH, which independently predicts poor outcomes, namely death and disability (8). Qin et al. investigated the association between leukocyte subpopulations and HE according to two definitions (Definition 1: volume increase ≥6 ml or 33%; Definition 2: volume increase ≥12.5 ml or 33%). The findings revealed that a higher monocyte count is associated with a higher risk of HE regardless of the two definitions, whereas an increased neutrophil count is associated with a decreased risk of HE according to Definition 1. Shi et al. retrospectively analyzed 105 patients with severe basal ganglia ICH and demonstrated that NLR was a better predictor of 30-day mortality than other risk factors. In addition, a systematic review and meta-analysis by Li J. et al. showed that higher low-density lipoprotein cholesterol (LDL-C) levels may reduce the risk of mortality in ICH patients. Elevated LDL-C levels are only inversely associated with 3-month mortality risks, not in-hospital mortality risks in these patients. The study by Coleman et al. enrolled 2,449 patients from the ERICH trial to reveal that the SAVED2 score, which is composed of several common predictors, is associated with unfavorable outcomes in patients with ICH. Finally, Li Z. et al. presented a review summarizing recent advances in therapeutic strategies and directions for ICH and discussing the barriers and issues that need to be overcome to improve the prognosis of ICH. Subarachnoid hemorrhage (SAH) is another devastating type of hemorrhagic stroke with high mortality and disability rates. Neuroinflammation is an important mechanism of injury after ICH (9). Zhang et al. included 3,173 aneurysmal SAH (aSAH) patients and compared the predictive effects of multiple inflammatory markers. The results indicated that one of these inflammatory markers, the neutrophil-to-albumin ratio (NAR) could improve the SAFIRE and SAHIT models for 3-month mortality. Furthermore, Ji et al. demonstrated that increased serum anion gap (AG) is an independent, significant, and robust predictor of all-cause in-hospital mortality. Venous thrombosis is another rare form of stroke. Zhou et al. contributed a review that provided a reference for a comprehensive understanding of venous thrombosis and a scientific understanding of various pathophysiological mechanisms and clinical features related to this condition.

Several articles in our Research Topic concern both ischemic and hemorrhagic stroke. Zhao et al. analyzed the data of 1,601 stroke patients and found that inflammatory biomarkers such as NLR, NAR, and the ratio of red cell distribution width to albumin (RA) were able to predict 30-day mortality in hemorrhagic stroke but not in ischemic stroke. Matuja et al. investigated the predictors of 30-day mortality in patients with stroke in northwestern Tanzania. Their findings suggested that NIHSS and mRS on admission, aspiration pneumonia, and electrocardiogram abnormalities were associated with 30-day mortality. Importantly, Zhu et al. used several highly interpretable machine learning models to predict stroke prognosis with the highest accuracy to date and to identify heterogeneous treatment effects of warfarin and human albumin in stroke patients.

Overall, the current Research Topic of 37 articles provides a comprehensive overview of the latest advances in this field. It includes neuroimaging and body fluid markers that predict stroke outcomes. Moreover, the articles also include the latest developments in medical and surgical therapies that improve stroke outcomes. These findings may provide new insights for clinical practice. Further efforts are needed to investigate more beneficial therapeutic strategies, perhaps with the help of effective predictors.

## Author contributions

HC contributed to the drafting and writing of the editorial. HC, LL, BQ, and YT were responsible for proofreading, editing, and reviewing the manuscript. All authors approved the version submitted for publication.
